# Comparison of Mortality Following Hospitalisation for Isolated Head Injury in England and Wales, and Victoria, Australia

**DOI:** 10.1371/journal.pone.0020545

**Published:** 2011-05-31

**Authors:** Belinda J. Gabbe, Ronan A. Lyons, Fiona E. Lecky, Omar Bouamra, Maralyn Woodford, Timothy J. Coats, Peter A. Cameron

**Affiliations:** 1 Department of Epidemiology and Preventive Medicine, Monash University, The Alfred Hospital, Melbourne, Victoria, Australia; 2 National Trauma Research Institute, The Alfred Hospital, Melbourne, Victoria, Australia; 3 School of Medicine, Swansea University, Singleton Park, Swansea, United Kingdom; 4 The Trauma Audit and Research Network, Health Sciences Research Group, School of Community Based Medicine, Manchester Academic Health Sciences Centre, University of Manchester, Salford Royal Hospital, Salford, United Kingdom; 5 Department of Emergency Medicine, Salford Royal Hospital, Salford, United Kingdom; 6 Accident and Emergency Department, Leicester Royal Infirmary, Leicester, United Kingdom; 7 Emergency and Trauma Centre, The Alfred Hospital, Melbourne, Victoria, Australia; Institute Biomedical Research August Pi Sunyer (IDIBAPS) - Hospital Clinic of Barcelona, Spain

## Abstract

**Background:**

Traumatic brain injury (TBI) remains a leading cause of death and disability. The National Institute for Health and Clinical Excellence (NICE) guidelines recommend transfer of severe TBI cases to neurosurgical centres, irrespective of the need for neurosurgery. This observational study investigated the risk-adjusted mortality of isolated TBI admissions in England/Wales, and Victoria, Australia, and the impact of neurosurgical centre management on outcomes.

**Methods:**

Isolated TBI admissions (>15 years, July 2005–June 2006) were extracted from the hospital discharge datasets for both jurisdictions. Severe isolated TBI (AIS severity >3) admissions were provided by the Trauma Audit and Research Network (TARN) and Victorian State Trauma Registry (VSTR) for England/Wales, and Victoria, respectively. Multivariable logistic regression was used to compare risk-adjusted mortality between jurisdictions.

**Findings:**

Mortality was 12% (749/6256) in England/Wales and 9% (91/1048) in Victoria for isolated TBI admissions. Adjusted odds of death in England/Wales were higher compared to Victoria overall (OR 2.0, 95% CI: 1.6, 2.5), and for cases <65 years (OR 2.36, 95% CI: 1.51, 3.69). For severe TBI, mortality was 23% (133/575) for TARN and 20% (68/346) for VSTR, with 72% of TARN and 86% of VSTR cases managed at a neurosurgical centre. The adjusted mortality odds for severe TBI cases in TARN were higher compared to the VSTR (OR 1.45, 95% CI: 0.96, 2.19), but particularly for cases <65 years (OR 2.04, 95% CI: 1.07, 3.90). Neurosurgical centre management modified the effect overall (OR 1.12, 95% CI: 0.73, 1.74) and for cases <65 years (OR 1.53, 95% CI: 0.77, 3.03).

**Conclusion:**

The risk-adjusted odds of mortality for all isolated TBI admissions, and severe TBI cases, were higher in England/Wales when compared to Victoria. The lower percentage of cases managed at neurosurgical centres in England and Wales was an explanatory factor, supporting the changes made to the NICE guidelines.

## Introduction

Head injury, or traumatic brain injury (TBI), remains a leading cause of death and disability worldwide with the majority of deaths related to severe TBI. Although TBI has been termed an “untreatable” predictor of mortality [1], advances in trauma care systems, pre-hospital care, and critical care have led to improvements in patient survival following TBI. There is evidence to suggest that integration of trauma services [2], [3], [4], direct transport of patients from the scene to hospital [5], and the level of pre-hospital care can impact on TBI patient survival [6].

The National Institute for Health and Clinical Excellence (NICE) guidelines were published in 2003 to provide evidence-based guidelines for the management of head injury in the United Kingdom (UK) (http://www.nice.org.uk/nicemedia/live/11836/36259/36259.pdf). These were updated in 2007 when the key recommendation of transfer of severe TBI cases to neurosurgical centres, irrespective of the need for neurosurgery, was added. This recommendation was supported by the results of a previous UK study which found a higher rate of mortality in TBI patients managed in non-neurosurgical centres compared to patients managed within neurosurgical centres from 1999–2003 [7]. However, the capacity of UK neurosurgical services to manage the volume of these cases has been questioned [8].

Management of TBI differs across health jurisdictions with respect to triage and referral, guidelines, and trauma care delivery but the impact on patient outcomes is unclear. Comparison of outcomes across health jurisdictions requires analysis of contemporaneous data collected using comparable methodology and data items. Trauma registry data and hospital discharge data represent two key sources of TBI surveillance. Trauma registries collect detailed injury event, severity and management data but usually only for a select group of injured patients and not all trauma registries have full population coverage. In contrast, hospital discharge datasets provide population-based data about all cases admitted to hospital but collect limited injury-specific data such as TBI severity and clinically relevant data items (e.g. Glasgow Coma Scale (GCS)) necessary for more detailed risk-adjustment.

This observational study used trauma registry and hospital admissions data from England and Wales and Victoria, Australia to compare the mortality outcomes of isolated head injuries across these health jurisdictions. Trauma registry data was used to: (i) establish the prevalence of neurosurgical centre management of *severe* isolated TBI in England and Wales, and Victoria, Australia; (ii) compare the risk-adjusted mortality of *severe* isolated TBI in England and Wales, and Victoria; and (iii) evaluate the impact of neurosurgical centre care on risk-adjusted mortality in severe TBI. Population-based hospital admissions data from both jurisdictions were used to compare the risk-adjusted mortality of all isolated TBI cases following admission to hospital in England and Wales, and Victoria, Australia and compare the findings with those obtained through trauma registry data analysis. This study focused on isolated TBI because it is common, has high mortality risk, and does not require the additional care needs associated with significant extracranial injuries.

## Methods

### Setting

Victoria is the second most populous state in Australia, comprising 5.3 million (4.1 million >15 years) people and 25% of the Australian population (www8.abs.gov.au). The population of England and Wales is approximately 52 million (43.5 million >15 years) (www.statistics.gov.uk). Both countries provide a high level of health care, with the percentage of gross domestic product spent on health care in 2007 similar across the countries (8.4% for the UK and 8.9% in Australia). Victoria operates an inclusive, regionalised trauma system, with seriously injured patients triaged to specialist major trauma service hospitals, a level of trauma care delivery not yet fully implemented in England and Wales.

### Datasets

Hospital discharge and trauma registry data were obtained from both jurisdictions for comparison.

#### Trauma Registries

Trauma registries provide detailed clinical information not captured by administrative datasets such as hospital discharge datasets. Serious isolated TBI cases were extracted from the UK's Trauma Audit and Research Network (TARN) database and the Victorian State Trauma Registry (VSTR). The TARN database commenced in 1989. Since 1996, TARN has been collecting information from approximately half of the trauma receiving hospitals in England and Wales (www.tarn.ac.uk/standardsofcare). The VSTR is a population-based registry, capturing information about all major trauma patients in Victoria since July 2001. The data collection methods of TARN and VSTR have been published previously [9], [10], [11] and both registries focus on the severe end of the injury spectrum including all injury-related in-hospital deaths. The VSTR data collection has been approved by the Monash University Human Research Ethics Committee, all 138 participating institutions and their relevant ethics committees.

Patients aged greater than 15 years, with an isolated, severe TBI, were extracted for analysis from the VSTR and TARN registries for the period July 2005 to June 2006. The Abbreviated Injury Scale (AIS) classification system classifies injuries by type and severity. For each diagnosis, a severity score on the scale 1 (minor) to 6 (maximum) is provided representing the risk of mortality. For this study, an isolated, severe TBI was defined as a head injury with an Abbreviated Injury Scale (AIS) severity score >3 (serious), and no associated injuries with an AIS severity score >1 (minor). Both registries used the 1990 revision of the AIS (1998 update) for the study period. As the GCS score is not a criterion for registry inclusion, selection of severe cases based on the GCS score was not possible. Comparable data items were extracted from the registries, including demographic data, injury event information, diagnoses, injury severity, observations on arrival to the definitive hospital of care, critical care management, and in-hospital outcomes. Cases transferred to a non-TARN participating hospital for definitive management were excluded as the final outcome could not be determined. Cases from VSTR who were discharged directly home within 72 hours of admission were excluded as these do not fulfil TARN criteria.

#### Hospital Discharge Data

A limitation of reliance on trauma registry data for the inter-jurisdictional comparison is that TARN does not have complete population coverage, raising the potential for selection bias to impact on the study findings. Therefore, hospital discharge data were sourced to enable population-based comparison of isolated TBI admissions and to compare the findings with the registry-based analyses. All head injury-related hospital admissions for the period July 2005 to June 2006 (inclusive) were extracted for analysis. Data for England were obtained from the Hospital Episode Statistics (HES) database which is the national statistical data warehouse for England provided by National Health Service (NHS) hospitals. Admissions data for Wales were obtained from the Patient Episode Database for Wales (PEDW) which captures data for all inpatient care provided at NHS hospitals in Wales. Data for Victoria were obtained from the Victorian Admitted Episodes Dataset (VAED) which contains morbidity data for all admitted patients to public and private hospitals in the state.

Hospital discharge datasets use the International Classification of Diseases (ICD) system, an international standard diagnostic classification for all diseases and injuries. England, Wales and Victoria use the 10^th^ revision, ICD-10. The ICD-10 system is not specific to injuries, and the diagnosis codes do not specifically specify injury severity. For example, the ICD-10 system has a single code for all traumatic subdural haemorrhages (S06.5) while the AIS system has four codes for subdural haemorrhages with each code representing a different size of haemorrhage and severity score.

In this study, for patients aged >15 years, emergency hospital admissions where the principal ICD-10 diagnosis was a skull fracture (S02.0, S02.1, S02.9) or intracranial injury (S06.1-S06.9) were extracted for analysis. Hospital admissions with a length of stay <24 hours (excluding deaths), or where the patient was discharged to another hospital (i.e. inter-hospital transfer) were excluded. Variables extracted for analysis included the age group, sex, length of stay, discharge destination and all ICD-10 diagnoses for the admission. Hospital discharge data do not include GCS or AIS data.

An admission was classified as an isolated head injury if the ICD-10 codes for the admission did not contain an ICD-10 S or T Chapter XIX code for a body region other than the head, excluding superficial injuries (S10–S10.9, S20–S20.9, S30–S30.9, S40–S40.9, S50–S50.9, S60–S60.9, S70–S70.9, S80–S80.9, S90–S90.9, or T00–T00.9). All in-hospital deaths were identified from the discharge destination. The mechanism of injury was identified from the first recorded ICD-10 Chapter XX code listed.

### Data analysis

Summary statistics were used to describe the profile of cases for England and Wales, and Victorian cases for each data source. Chi-square tests were used to assess the association between population descriptors and health jurisdiction for categorical variables, and either Mann-Whitney U tests or independent t-tests were used for continuous variables. Multivariable logistic regression was used to determine the association between health jurisdiction and outcome (in-hospital mortality) after adjusting for differences in the case-mix across the jurisdictions (variables with a p-value <0.05) and known predictors of mortality, for hospital discharge and trauma registry data. Separate models were generated for hospital discharge data and trauma registry data. Models were developed for all cases and for those aged <65 years, with the latter group important as it excludes the potential complexities of acute on chronic episodes of head injury that are common in the elderly, and represents the group most likely to benefit from advanced trauma care [12], [13]. Because of the importance of the GCS as a predictor of mortality following head injury, and the prevalence of missing GCS data across the registries, the missing GCS scores were imputed using multiple imputation by chained equations, and included in the trauma registry analyses. The method used to impute the GCS has been previously described [14]. Adjusted odds ratios (AOR) and 95% confidence intervals (95% CI) were calculated. An a priori p-value <0.05 was considered significant.

## Results

### Trauma registry data

There were 346 severe, isolated TBI cases captured by the VSTR, and 575 by TARN, over the 12-month period. Cases from TARN were more commonly male and younger than VSTR cases (Table 1). Consistent with the age difference, a higher percentage of VSTR cases were the result of a fall while the TARN cases were more severely injured according to the AIS severity score and the GCS (Table 1). Comparisons based on physiological observations were limited by the high volume of missing data for some variables (Table 1 footnote).

**Table 1:** Comparison of the 2005-06 patient profile of severe isolated traumatic brain injury from England and Wales (TARN) and Victoria, Australia (VSTR).

Variable			VSTR	TARN	p-value
			(n = 346)	(n = 575)	
**Age (years)**	Mean (SD)		59.0 (23.4)	49.2 (22.0)	<0.0001
**Gender**	n (%)				
	Male		235 (67.9)	433 (75.3)	0.015
	Female		111 (32.1)	142 (24.7)	
**Cause of injury**	n (%)				
	Falls		237 (69.5)	321 (55.8)	<0.001
	Transport-related		38 (11.1)	103 (17.9)	
	Other cause		66 (19.4)	151 (26.3)	
**On arrival at ED** a	Pulse rate	Mean (SD)	84.4 (19.1)	83.5 (22.5)	0.540
	Systolic BP	Mean (SD)	148.0 (29.8)	145.7 (29.3)	0.295
	Respiratory rate	Mean (SD)	17.6 (3.9)	18.6 (17.0)	0.041
	O_2_ saturation	Median (IQR)	99 (97–100)	99 (97–100)	0.219
	GCS^b^ score	n (%)			
		Severe (3–8)	92 (27.2)	130 (34.7)	0.006
		Moderate (9–12)	34 (10.1)	54 (14.4)	
		Mild (13–15)	212 (62.7)	191 (50.9)	
**Highest head injury severity (AIS** c **)**	n (%)				
	4		217 (62.7)	294 (51.1)	0.001
	5–6		129 (37.3)	281 (48.9)	
**Inter-hospital transfer?** d	n (%)				0.716
	No		204 (59.0)	346 (60.2)	
	Yes		142 (41.0)	229 (39.8)	
**Managed at a neurosurgical centre?**	n (%)				<0.001
	No		50 (14.5)	161 (28.0)	
	Yes		296 (85.5)	414 (72.0)	
**Critical care stay?**	n (%)				0.019
	No		240 (69.4)	355 (61.7)	
	Yes		106 (30.6)	220 (38.3)	
**Hospital length of stay**	Median (IQR)		7 (4–13)	7 (4–16)	0.181
**In-hospital outcome**	n (%)				0.216
	Survived		274 (80.4)	442 (76.9)	
	Died		68 (19.6)	133 (23.1)	

a ED - emergency department; VSTR data missing for pulse (2.3%), SBP (2.0%), GCS (2.3%), O_2_ saturation (3.7%) and respiratory rate (12.7%). TARN data missing for 33.7% (SBP), 35.5% (pulse), 34.8% (GCS), 49.6% (respiratory rate) and 45.4% (O_2_ saturation);

b GCS, Glasgow Coma Scale;

c AIS, Abbreviated Injury Scale;

d TARN cases transferred to a non-TARN participating hospital were excluded.

A higher percentage of cases captured by TARN required a critical care unit stay (Table 2). Only 72% of TARN cases were definitively managed in a neurosurgical centre compared to 86% of VSTR cases (Table 1). The crude in-hospital mortality rate in the UK was 3.5% higher than for Victoria, Australia (Table 1) with TARN cases demonstrating a non-significant elevated unadjusted odds of death compared to Victoria (OR 1.23, 95% CI: 0.90, 1.71). Adjusting for the differences in case-mix (age, gender, head injury severity, cause of injury, and the GCS), cases in the UK were at elevated odds of mortality compared to Victoria, Australia (Table 2), though just failing to reach significance. The addition of management at a neurosurgical centre to the multivariate model modified the effect of trauma setting on mortality (AOR 1.12; 95% CI: 0.73, 1.74).

**Table 2:** Association between trauma setting and in-hospital mortality for cases with a severe isolated traumatic brain injury –multivariable analysis for all cases (n = 921) and cases aged <65 years (n = 592).

Variable		All cases	Cases aged <65 years
		AOR^a^ (95% CI)	AOR (95% CI)
**Trauma setting**	VSTR^b^ (reference)	1.00	1.00
	TARN^c^	1.45 (0.96, 2.19)	2.04 (1.07, 3.90)
**Age (years)**		1.04 (1.03, 1.05)	1.03 (1.01, 1.06)
**Gender**	Male (reference)	1.00	1.00
	Female	1.03 (0.65, 1.63)	0.76 (0.37, 1.58)
**Cause of injury**	Transport-related (reference)	1.00	1.00
	Falls	1.23 (0.69, 2.19)	1.69 (0.80, 3.26)
	Other	0.57 (0.26, 1.21)	0.68 (0.28, 1.62)
**GCS** d	Mild (13–15) (reference)	1.00	1.00
	Moderate (9–12)	4.46 (2.30, 8.67)	2.45 (0.72, 8.29)
	Severe (3–8)	11.68 (6.39, 21.33)	12.12 (5.64, 26.06)
**Head injury severity (AIS** e **severity score)**	4 (reference)	1.00	1.00
	5–6	3.27 (2.15, 4.96)	3.05 (1.68, 5.53)

a AOR, Adjusted odds ratio;

b VSTR, Victorian State Trauma Registry;

c TARN, Trauma Audit and Research Network;

d GCS, Glasgow Coma Scale;

e AIS, Abbreviated Injury Scale.

Repetition of the analysis with older adults excluded provided more pronounced findings. The percentage of cases aged <65 years managed at a neurosurgical centre was 76% for TARN and 92% for VSTR cases; the in-hospital mortality was 17% (68/405) for TARN and 9.8% (17/173) for VSTR cases overall. The mortality rate for cases managed at a neurosurgical centre was 16% for TARN and 16% for the VSTR, while 40% of TARN, and 30% of VSTR, cases managed at a non-neurosurgical centre died during their hospital stay. The unadjusted odds of death for isolated severe head injury cases aged <65 years in the TARN cases was 1.85 (1.05, 3.26) compared to VSTR cases. The case-mix adjusted OR (95% CI) was 2.04 (1.07, 3.90) (Table 2), and 1.53 (0.77, 3.03) when the variable pertaining to management at a neurosurgical centre was included.

### Hospital discharge data

There were 6,256 isolated TBI admissions in England and Wales, and 1048 in Victoria, in 2005-06. The much higher number of isolated TBI cases in the hospital discharge data reflects the reliance on ICD-10 diagnosis codes to select the cases, as identified in the methods. There were differences in case-mix across the jurisdictions with respect to age, mechanism of injury and principal diagnosis, with a higher percentage of admissions in the 85 years and over group for England and Wales (Table 3). The percentage of cases in the “other/not further specified” categories for principal diagnosis and mechanism of injury were higher for England and Wales (Table 3). The ICD-10 cause code was missing for 548 (8.8%) of admissions for England and Wales compared to 19 (1.8%) cases for Victoria. The profile of road trauma cases was consistent across the jurisdictions except for a higher proportion of motor vehicle collisions in Victoria, and a lower proportion of pedestrian and pedal cyclist incidents, compared to England and Wales. Consistent with the trauma registry data analysis, the in-hospital mortality rate for England and Wales was higher (Table 3), and the unadjusted odds of death for all isolated TBI admissions was 1.43 (95% CI: 1.14, 1.80) times higher than for Victoria. After adjusting for the mechanism of injury, age, gender, and principal head injury diagnosis, the odds of mortality remained significantly higher for England and Wales compared to Victoria (OR 1.98, 95% CI: 1.56, 2.52) (Table 4).

**Table 3:** Profile of isolated traumatic brain injury admissions in England and Wales, and Victoria, Australia (2005-06).

Population descriptor		England and Wales(n = 6256)	Victoria, Australia(n = 1048)	p-value
**Sex**	n (%)			p = 0.275
	Male	4420 (70.7)	723 (69.0)	
	Female	1836 (29.3)	325 (31.0)	
**Age group**	n (%)			p<0.001
	15–24 years	1087 (17.4)	172 (16.4)	
	25–34 years	841 (13.4)	145 (13.8)	
	35–44 years	792 (12.7)	114 (10.9)	
	45–54 years	653 (10.4)	97 (9.3)	
	55–64 years	619 (9.9)	99 (9.4)	
	65–74 years	531 (8.5)	108 (10.3)	
	75–84 years	743 (11.9)	203 (19.4)	
	≥85 years	990 (15.8)	110 (10.5)	
**Mechanism of injury**	n (%)			p<0.001
	Falls	3286 (52.5)	580 (55.3)	
	Assault	1134 (18.1)	193 (18.4)	
	Transport-related	902 (14.4)	162 (15.5)	
	Animate or inanimate mechanical forces	258 (4.1)	79 (7.5)	
	Other/not further specified	676 (10.8)	34 (3.2)	
**Principal diagnosis**	n (%)			p<0.001
	Skull fracture	1698 (27.1)	216 (20.6)	
	Traumatic subdural haemorrhage	1592 (25.5)	399 (38.1)	
	Traumatic extradural haemorrhage	350 (5.6)	57 (5.4)	
	Traumatic subarachnoid haemorrhage	466 (7.4)	114 (10.9)	
	Diffuse brain injury	574 (9.2)	114 (10.9)	
	Focal brain injury	317 (5.1)	99 (9.5)	
	Other/not further specified intracranial injury	1259 (20.1)	49 (4.7)	
**Hospital length of stay**	Mean (SD) days	7.42 (15.43)	7.28 (21.20)	p = 0.806
**In-hospital mortality**	n (%)			p = 0.002
	Survivor	5507 (88.0)	957 (91.3)	
	Death	749 (12.0)	91 (8.7)	

**Table 4:** Association between country and mortality in isolated traumatic brain injury admissions – multivariable analysis for all cases (n = 7304) and cases aged <65 years (n = 4619).

Variable		All casesAOR^a^ (95% CI)	Cases aged <65 yearsAOR (95% CI)
**Country**	Victoria, Australia (reference)	1.00	1.00
	England and Wales	1.98 (1.56, 2.52)	2.36 (1.51, 3.69)
**Sex**	Male (reference)	1.00	1.00
	Female	0.87 (0.74, 1.03)	0.71 (0.53, 0.95)
**Age**	15–24 years (reference)	1.00	1.00
	25–34 years	1.11 (0.73, 1.68)	1.12 (0.74, 1.69)
	35–44 years	1.21 (0.81, 1.80)	1.24 (0.83, 1.85)
	45–54 years	2.06 (1.41, 2.99)	2.14 (1.46, 3.15)
	55–64 years	1.78 (1.22, 2.60)	1.83 (1.24, 2.71)
	65–74 years	2.67 (1.84, 3.87)	-
	75–84 years	3.62 (2.54, 5.14)	-
	≥85 years	3.26 (2.28, 4.67)	-
**Mechanism**	Falls (reference)	1.00	1.00
	Assault	0.32 (0.22, 0.47)	0.35 (0.23, 0.52)
	Road trauma	0.97 (0.75, 1.24)	1.19 (0.87, 1.63)
	Animate or inanimate mechanical forces	0.39 (0.23, 0.69)	0.25 (0.10, 0.69)
	Other/not further specified	0.79 (0.61, 1.03)	0.99 (0.69, 1.41)
**Principal diagnosis**	Skull fracture (reference)	1.00	1.00
	Traumatic subdural haemorrhage	7.34 (5.35, 10.07)	9.53 (5.85, 15.53)
	Traumatic extradural haemorrhage	3.53 (2.17, 5.73)	3.11 (1.59, 6.11)
	Traumatic subarachnoid haemorrhage	8.56 (5.94, 12.32)	7.84 (4.55, 13.52)
	Diffuse brain injury	7.02 (4.85, 10.15)	6.35 (3.74, 10.80)
	Focal brain injury	3.85 (2.43, 6.13)	3.61 (1.84, 7.10)
	Other/not further specified intracranial injury	3.56 (2.51, 5.05)	3.32 (1.95, 5.67)

a AOR, adjusted odds ratio.

Similar to the trauma registry findings, for cases aged <65 years, the in-hospital mortality rate was lower in Victoria (23/627, 3.7%) compared to England and Wales (300/3992, 7.5%) with the unadjusted odds of mortality significantly higher in England and Wales (2.13, 95% CI: 1.38, 3.29). After adjusting for case-mix differences, the odds of mortality remained significantly higher for the young, isolated head injury admissions in England and Wales compared to Victoria (Table 4).

## Discussion

Two data sources were used to explore the outcomes of hospitalised, isolated TBI in England and Wales, and Victoria, Australia; jurisdictions with different levels of organisation of trauma care delivery. Using trauma registry data, the odds of dying following hospitalisation for a severe, isolated TBI were higher in England and Wales when compared to Victoria, particularly for younger adults. When population-based hospital discharge data were used to assess mortality outcomes for all hospital admissions following isolated TBI, the findings were consistent with increased odds of death in England and Wales overall, and for cases aged <65 years.

The percentage of cases managed at a neurosurgical centre was significantly lower in the England and Wales data, and appeared to be a key explanatory factor for the differences in mortality observed. Patel *et al* reported that 67% of patients with severe TBI in the UK were definitively managed in a neurosurgical centre between 1999 and 2003, and that only 53% of cases arriving at a non-neurosurgical centre were transferred to a neurosurgical centre [7]. On the basis of their findings, Patel *et al* argued for transfer of all severe TBI patients to hospitals with 24-hour neurosurgical services [7], a recommendation added to the updated NICE guidelines in 2007. In the current study, the percentage of severe TBI cases managed in neurosurgical centres in England and Wales was higher, reaching 72% for all adult cases and 76% of those aged <65 years. However, the rates were significantly lower when compared to Victoria (86% for all adults and 92% for those <65 years), where the pre-hospital triage and system guidelines dictate the transport of major trauma patients to a major trauma service (Level 1 trauma centre) which all have neurosurgical services, and a selection of isolated TBI cases to alternative neurosurgical services. Overall, the mortality rate for severe, isolated TBI (AIS severity score >3) cases managed at neurosurgical centres was much lower than cases managed at non-neurosurgical centres for England and Wales (16% vs. 40%) and Victoria (16% vs. 30%).

The findings suggest that improvement in the transport of severe TBI patients to an appropriate facility in the UK could reduce mortality following severe, isolated TBI. However, the issue of neurosurgical service availability remains. Esposito and colleagues argued that the availability of neurosurgical services at all Level 1 trauma centres, an essential verification item, may not be feasible given training and attrition rates of neurosurgeons in the US [15], a country considered to be well resourced for healthcare [8]. In Victoria, there are 138 designated trauma receiving hospitals, three major trauma services (each with neurosurgical services), and three further neurosurgical centres, for a population of 5.3 million. The population of the UK is approximately 10 times the Victorian population, and there are 298 district general hospitals with emergency departments and 35 neurosurgery units [8]. These figures confirm the concerns expressed by Mendelow *et al* that additional resources would be needed to staff and equip the neurosurgical services appropriately for managing the volume of severe TBI cases in the UK.

The most appropriate delivery of trauma services and neurosurgical care has stimulated considerable research and debate. There is a growing body of evidence that integrated trauma systems improve outcomes for trauma patients [2], [16], [17], [18], [19], [20]. In the case of the multi-trauma patient with associated TBI (AIS head injury severity score >2 (moderate)), outcomes were significantly better over a five-year period in the presence of an integrated trauma system in Victoria, Australia when compared to England and Wales where such systems are not well developed [21]. Management at a neurosurgical centre did not account for the difference in outcomes observed [21]. In contrast, in the current study focusing on isolated, severe TBI (AIS severity score >3 (serious)), management at a neurosurgical centre was an important factor. Together, these findings suggest that the multi-trauma patient involving TBI has different needs for care when compared to the isolated TBI group. The selection of facilities for management is more complex for the multi-trauma patient than simply transfer to a neurosurgical centre, a point highlighted in the 2007 update of the NICE guidelines. The redevelopment of trauma services and delivery in the UK is currently underway and should contribute to improved triage and referral of cases to appropriate care facilities.

The current study was able to use comparable and contemporaneous clinical and administrative data to compare the outcomes of TBI admissions across health jurisdictions, but there were a number of study limitations. Although care at a neurosurgical centre appeared to be protective against mortality in this study, the study design was unable to establish a causal relationship and studies capable of establishing causation (e.g. randomised controlled trials) would not be feasible. It is possible that other potential, unmeasured confounders could contribute to the study findings. Direct comparison of neurosurgical intervention rates in the VSTR and TARN were limited as the method employed by TARN to collect the data led to difficulties in determining whether an unpopulated field represented the absence of the event (e.g. craniotomy) or missing data. The use of intracranial pressure (ICP) monitoring and neurosurgical intervention were two of these variables. Nevertheless, the recorded percentage of cases undergoing craniotomy was 28% for VSTR and 30% for TARN. For ICP monitoring, it was 8.1% for VSTR and 8.9% for TARN. The mortality rate for patients managed at neurosurgical centres where no neurosurgical intervention was recorded was similar for the VSTR (14%) and TARN (17%). Overall, there was little difference in the rates of neurosurgical intervention across the settings, suggesting that the importance of neurosurgical centre care is not fully explained by the use of neurosurgical interventions.

The VSTR is a population-based registry but participation in TARN is voluntary and not complete for all trauma receiving hospitals, raising the potential for selection bias in the TARN dataset. Cases transferred to hospitals not participating in TARN were also excluded as the outcomes for these patients were unknown, a source of potential bias. While the bias is believed to be towards higher volume, better performing hospitals participating in TARN, the impact of the lower coverage by TARN on the estimate of mortality risk cannot be definitively determined. To address this potential bias, population-based discharge data were used to compare outcomes across both settings for isolated TBI admitted to hospital to see if the findings using registry data were consistent with the findings of analysis of hospital discharge data. The consistent findings of higher risk-adjusted mortality in England and Wales compared to Victoria suggest that the differences found using the registry data are unlikely to be due to TARN selection bias alone and there is evidence of real differences in mortality outcomes between England and Wales, and Victoria.

Nevertheless, although both hospital discharge data and trauma registry data were able to be obtained, and provided consistent findings, the classification of head injury was not consistent across the datasets. The NICE guidelines recommend the classification of TBI severity based on the GCS score, a scoring system based on the level of consciousness. The GCS is not an inclusion criterion for entry onto the TARN or VSTR databases, is commonly missing in trauma registries [14], [22], and is difficult to collect in cases affected by drugs and alcohol [22], [23]. Therefore, cases were identified according to the AIS classification system, a system that classifies head injury severity based on anatomical injury. The analyses were adjusted for GCS score where available and the combination of AIS and GCS has been shown to improve prediction of outcome over GCS alone [24].

The hospital discharge datasets do not collect AIS or GCS data and therefore case selection was based on the ICD-10 system. The ICD-10 system does not include measures of severity comparable to AIS, but attempts were made to limit the discharge data to the intracranial injuries and skull fractures likely to qualify as serious head injuries under the AIS system. There was a high prevalence of “unspecified” intracranial injury coding in the HES and PEDW datasets, compared to the VAED, which could highlight differences in diagnostic practices across the countries and limited the quality of the risk-adjustment analysis. Preliminary registry data suggest lower head CT scan rates for UK cases compared to Victorian cases (62% vs. 84%), which could explain the higher prevalence of specific diagnosis codes in the Victorian discharge data. Similarly, the prevalence of “unspecified” mechanism of injury codes was higher for the UK data, somewhat limiting the risk adjusted analysis.

The observation that the majority of isolated TBI admissions in both countries were not included in the registries reflects the selection of registry cases for this study. For the registry analyses, we included isolated TBI cases with an AIS severity score >3, which excludes simple skull fractures and any cases of concussion with a normal CT brain that may have been coded as brain injury at hospital discharge in routine data. The proportion of HES/PEDW admissions included in TARN appears low at 9% compared to the 33% included by the VSTR. There are several reasons for this including a relatively low TARN membership (40% of trauma receiving hospitals during the study period), and 2677 cases in the HES/PEDW dataset meeting the VSTR criteria but not the TARN criteria. These were: (a) patients who stayed in hospital less than 3 days and did not die (1748 cases); and (b) elderly patients with likely chronic SDHs (mechanism absent) where we cannot be sure that the cause of the SDH was traumatic (929 cases). Removing the cases with a stay length of less than 3 days from the calculation gives a capture rate of 13%. Removing the elderly SDH cases as well gives a TARN capture rate of 16%, approximating the VSTR's coverage.

In addition, the prevalence of missing data for physiological observations was high, limiting the ability to compare the clinical stability of patients across the settings. In particular, the prevalence of missing GCS data, a key predictor of mortality following TBI, was a barrier to analysis. Multiple imputation methods were used to address the missing GCS data and avoid the biases associated with complete case analysis (inclusion of cases only with complete GCS data) [14], but there is no clear consensus about the best method for imputation and these methods will always be inferior to analysis based on complete primary data collection. Multiple imputation assumes the GCS scores were missing at random which cannot be completely ascertained and previous studies have shown that the method of imputation can substantially change the overall findings [25]. Overall, the results were consistent whether using imputation techniques or complete cases analysis except for less precision of the estimates for the complete case analyses related to lower case numbers included. Using complete case analysis, the adjusted odds of mortality overall, and for cases <65 years, were 2.11 (95% CI: 1.33, 1.36) and 2.92 (95% CI: 4.43, 5.98), respectively. Addition of the variable related to neurosurgical centre care also produced similar results to the imputation-based models for all cases (adjusted OR 1.53, 95% CI: 0.92, 2.53), and cases aged <65 years (adjusted OR 1.89, 95% CI: 0.87, 4.11).

The outcome of interest in this study was mortality following hospitalisation for isolated TBI. Many TBI patients survive their injuries and the quality of survival is as important as survival [26]. While the VSTR routinely captures long term functional and health-related quality of life outcomes for all patients [27], these outcomes are not yet routinely collected by TARN, precluding comparison.

Overall, the odds of dying following hospitalisation for an isolated TBI were significantly higher in England and Wales when compared to Victoria, Australia, despite adjustment for key confounders. The percentage of severe, isolated TBI cases managed at a neurosurgical centre was significantly lower in England and Wales, and appeared to be a key explanatory factor for the mortality differences observed. The findings support the need to transport severe, isolated TBI cases to neurosurgical centres. While this study pre-dates the NICE guidelines update, the findings of this study support the changes made to the NICE guidelines but also raise questions about the ability of UK neurosurgical services to provide the clinical response needed for optimal treatment of TBI patients. Future studies should consider comparison of data following implementation and uptake of the latest update of the NICE guidelines, and outcomes other than mortality such as longer term function.
